# The Association between Serum Vitamin D Concentration and New Inflammatory Biomarkers—Systemic Inflammatory Index (SII) and Systemic Inflammatory Response (SIRI)—In Patients with Ischemic Heart Disease

**DOI:** 10.3390/nu14194212

**Published:** 2022-10-10

**Authors:** Ewelina A. Dziedzic, Jakub S. Gąsior, Agnieszka Tuzimek, Marek Dąbrowski, Piotr Jankowski

**Affiliations:** 1 Medical Faculty, Lazarski University in Warsaw, 02-662 Warsaw, Poland; 2 Department of Internal Medicine and Geriatric Cardiology, Centre of Postgraduate Medical Education, 01-813 Warsaw, Poland; 3 Department of Pediatric Cardiology and General Pediatrics, Medical University of Warsaw, 02-091 Warsaw, Poland; 4 Department of Cardiology, Bielanski Hospital, 01-809 Warsaw, Poland; 5 Department of Epidemiology and Health Promotion, School of Public Health, Center of Postgraduate Medical Education, 01-826 Warsaw, Poland

**Keywords:** systemic inflammatory index, systemic inflammatory response index, vitamin D, ischemic heart disease, acute coronary syndrome

## Abstract

The incidence of ischemic heart disease (IHD) increases every year. This cardiovascular disease has an inflammatory factor in its etiology due to different immune cells that influence atherogenesis. New inflammatory biomarkers—the Systemic Inflammatory Index (SII) and the Systemic Inflammatory Response (SIRI)—attempt to describe the pro- and anti-inflammatory balance and quantify the complex impact of the immune system on atherosclerosis, while vitamin D has a multidirectional impact on the human body, including the cardiovascular and immune systems. Hence, the objective of this research was to analyze the association between SII and SIRI and serum vitamin D concentrations in patients with IHD. A significant correlation was observed between SIRI and 25(OH)D in the whole group and between both biomarkers (SII and SIRI) and 25(OH)D in the group of patients with ACS but not in the group of patients with stable IHD. The role of vitamin D in IHD complications and its association with new inflammatory biomarkers requires further well-designed, large-scale research.

## 1. Introduction

Cardiovascular diseases (CVD) are responsible for a third of deaths in the world [1]. Among CVDs, ischemic heart disease (IHD) is the leading cause of premature death [2]. In the past 30 years, a constant increase in the incidence of IHD has been observed [3] as a result of an aging society [4] with a higher frequency of CVD risk factors [5]. The main cause of IHD is atherosclerosis, a chronic inflammatory disease of the vessel walls with an accumulation of lipids in the intima [6]. The imbalance between pro- and antiatherogenic immune cells leads to the clinical manifestations of IHD—acute coronary syndrome (ACS) and ischemic cardiomyopathy [7]. 

Due to the inflammatory etiology of atherosclerosis, the increasing significance of common inflammatory markers is derived from their association with the increased risk of CVD [8,9], ACS [10,11,12,13], and all-cause mortality [14,15]. Taking into account the interactions between different immune cell lines and their functions in atherogenesis, two-line inflammatory indices—the platelet-lymphocyte ratio (PLR), neutrophil-lymphocyte ratio (NLR), and monocyte-lymphocyte ratio (MLR)—were described to correlate well with CVD and mortality risk [16,17,18]. New biomarkers using three cell lines, the systemic inflammatory response index (SIRI), and the systemic inflammatory reaction index (SII) were previously evaluated in the determination of the outcome of neoplastic diseases [19,20]. In cardiology, high SIRI was correlated with an increased risk of ACS in patients with chest pain [21], supraventricular tachycardia in patients with stroke [22], and major adverse cardiovascular events (MACE) in patients after ACS treated with percutaneous intervention (PCI) [23]. SII was associated with an increased risk of MACE in patients with heart failure [24], after cardiosurgery [25,26,27,28], and after PCI [23,29]. It was also described to be correlated with collateral circulation development [30], contrast-induced nephropathy [31,32], massive pulmonary thrombosis risk [33], and coronary artery disease diagnosis and its severity [21,34,35,36]. 

Vitamin D, a steroid hormone that regulates calcium homeostasis, has several functions in the immune system [37], cardiovascular system [38], and other systems [39]. In the immune system, it regulates the response through the vitamin D receptor (VDR) present in most immune cells by influencing gene transcription [40]. In addition, these cells can convert calcifediol to calcitriol, enabling the autoregulation of active vitamin D concentration in localized inflammation independently of systemic levels [41]. Calcitriol stimulates monocyte proliferation and differentiation and reduces the immune response through macrophage activation [42]. Recently, data from the vitamin D stimulation of lymphocytes and monocytes revealed 15 target genes for this hormone, as well as innate and adaptive response mediators that play a key role in the immune system [37]. In neutrophils, vitamin D modulates the immune response by blocking pro-inflammatory mediators and the production of reactive oxygen species (ROS) [43]. 

The discovery of VDR [44,45] and 1-α-hydroxylase [46] in cardiovascular cells indicated that calcitriol is involved in the regulation of the circulatory system. Animal models revealed that vitamin D regulates vessel wall tension [47] and prevents hypertrophy of the heart muscle [48]. Observational research has shown that this hormone influences the classic risk factors for CVD, such as hypertension [49], diabetes mellitus [50], dyslipidemia [51], and obesity [52]. Furthermore, calcitriol was documented to have an influence on the atherosclerotic process [53,54] and its clinical complications—ACS [55] and stroke [56]. Vitamin D deficiency below 15 ng/mL was reported to cause a twofold increase in the risk of ACS compared with levels exceeding this value [57,58], and after ACS, low serum vitamin D levels were correlated with an increased risk of MACE [59].

Taking into account the results of our recent article showing significantly higher SIRI and SII in patients with ACS [21] and lower serum calcitriol levels in this cohort compared with patients with stable IHD [60], the main objective of this research was to assess the association of these new biomarkers with serum calcitriol.

## 2. Materials and Methods

### 2.1. Population

A detailed description of the study population characteristics is presented elsewhere [21]. Results of patients who underwent coronary angiography due to suspected ACS and were treated with acetylsalicylic acid and atorvastatin or rosuvastatin were analyzed. The exclusion criteria were elevated erythrocyte sedimentation rate, serum C-reactive protein concentration >5 mg/L, total white blood count exceeding 1.0 × 10^4^ cells/μL, diagnosed active viral or bacterial infection, active neoplastic processes or paraneoplastic syndromes, chronic kidney disease (stages III–V), calcium and phosphorus metabolism disorders, and vitamin D ingestion as a dietary supplement or medication. Only patients who agreed to participate in the study in writing were included in this investigation.

### 2.2. Clinical Data

Data regarding the weight, height, and comorbidities of patients enrolled in this study were retrieved from electronic patient files. Obesity and overweight were diagnosed using body mass index calculations and the World Health Organization criteria [61]. According to 2021 European Society of Hypertension practice guidelines, hypertension was diagnosed if blood pressure exceeded an in-office measurement of 140/90 mmHg [62]. Hyperlipidemia was diagnosed if the patient did not meet the treatment targets set for their risk level based on the 2019 ESC/EAS guidelines for the management of dyslipidemias [63]. The 2019 ESC guidelines on diabetes, pre-diabetes, and cardiovascular disease criteria were used to diagnose diabetes [64]. 

Coronary angiography was performed by access through the radial or femoral artery to diagnose IHD on the basis of the existence of significant stenosis in the coronary arteries, and PCI was eventually performed if necessary [65]. ACS diagnosis was based on the criteria of the European Society of Cardiology guidelines, which are as follows: the increased concentration of markers of myocardial injury that occurs with the coexistence of at least one of the items mentioned here: results of imaging tests depicting myocardial necrosis or coronary artery thrombus identification on coronary angiography, changes in ECG suggesting ischemia, or symptoms of stenocardia [66].

### 2.3. Laboratory Data

Blood samples were obtained by cephalic vein venipuncture and analyzed in the hospital laboratory within two hours of collection. SII, defined as (neutrophil count) × (platelet count)/(lymphocyte count), and SIRI, defined as (neutrophil count) × (monocyte count)/(lymphocyte count), were calculated from the total blood count measured using an automatic blood counter. 

Serum vitamin D concentrations (1 ng/mL = 2.5 nmol/L) were evaluated with the DiaSorin LIAISON^®^ 25 OH Vitamin D TOTAL Assay (Stillwater, MN, USA). This chemiluminescent immunoassay (detection range 4–150 ng/mL, precision 5.0% CV, SD of precision 1.2% [67]) has a good agreement strength with the Elecsys Vitamin D Total Assay, previously approved for clinical use by the Endocrine Society reference values for vitamin D deficiency [68,69,70,71]. The results were classified according to the Endocrine Society’s guidelines of clinical practice for vitamin D deficiency: concentrations of <10 ng/mL were labeled as severe deficiency, concentrations between ≥10 and <20 ng/mL were labeled as moderate deficiency, concentrations between ≥20 and <30 ng/mL were labeled as a mild deficiency, and concentrations of ≥30 mg/mL were labeled as optimal concentrations [68]. 

### 2.4. Statistical Analysis

The data distribution was determined using a Shapiro–Wilk test. The relationship between the selected variables was analyzed with the Spearman correlation coefficient (R). The Mann–Whitney U test was used to study the differences in 25(OH)D between patients with stable IHD and ACS. Variables that were not normally distributed were log transformed (ln) for multiple regression analysis. The potential determinants for the magnitude of the 25(OH)D concentration were investigated using multiple regression analysis. A backward stepwise regression analysis was used to identify significant predictors of the 25(OH)D concentration from the independent variables. Binary logistic regression was employed to identify factors associated with the outcome variable. The model fitness was checked by using the Hosmer–Lemeshow goodness of fit test. To express the performance of the logistic regression models, the area under the curve (AUC) statistic was used. The two-sided significance level of *p* ≤ 0.05 was considered significant. Analyses were performed using Statistica 13.3 software 109 (TIBCO Software Inc., Palo Alto, Santa Clara, CA, USA).

## 3. Results

The results section is divided into five sub-sections: (1) general information about the study participants; (2) vitamin D concentrations data and the factors that might influence the serum 25(OH)D; (3) the correlation between 25(OH)D and inflammatory biomarkers; (4) differences in 25(OH)D, SII, and SIRI between stable IHD and ACS; and, finally, (5) multivariate logistic regression analysis to identify factors associated with ACS diagnosis.

### 3.1. Characteristics of Participants

A comprehensive description of the patients’ characteristics is presented in Table 1 (data are presented as numbers (%) or medians (interquartile ranges)) and elsewhere [21]. 

### 3.2. Determinants of 25(OH)D Concentration

We observed seasonal concentration deviations due to changes in the UVB availability in the sunlight in Warsaw, Poland (52°13′ N, 21°02′ E)—patients examined between May and October had higher vitamin D concentrations compared with those examined between November and April due to the UVB-dependent synthesis of vitamin D in the skin that occurs only from May to October at this latitude [72]. The median serum 25(OH)D level in the entire study group was 15.1 ng/mL (range: 4.0–55.0 ng/mL). The determinants of the ln25(OH)D concentration are presented in Table 2. The proposed model was significant and explained 7% of 25(OH)D variance (R^2^ = 0.069, F = 6.450, *p* < 0.001). A backward stepwise regression analysis revealed that the examination date and hyperlipidemia were the strongest determinants of 25(OH)D (*p* < 0.001 for both, R^2^ = 0.058, F = 16.179, *p* < 0.001). 

### 3.3. Correlation between SII, SIRI, and 25(OH)D Concentration in the Whole Group and Separately for Patients with Stable IHD and ACS

**Figure 1:**
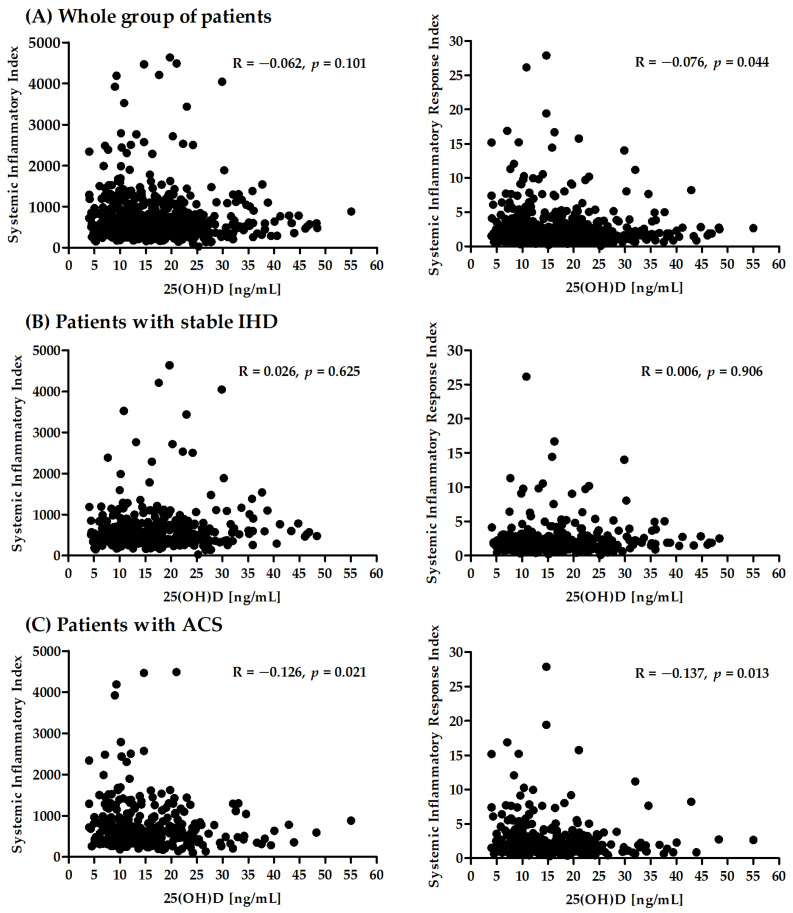
Correlation between SII, SIRI, and 25(OH)D concentration in the whole group (**A**) and separately for patients with stable IHD (**B**) and ACS (**C**).

In the present study, a correlation analysis between SII and SIRI and serum vitamin D concentrations in patients with IHD was performed. A significant correlation was observed between SIRI and 25(OH)D in the whole group and between both biomarkers and 25(OH)D in the group of patients with ACS (Figure 1). 

### 3.4. Differences in 25(OH)D, SII, and SIRI between Stable IHD and ACS

**Figure 2:**
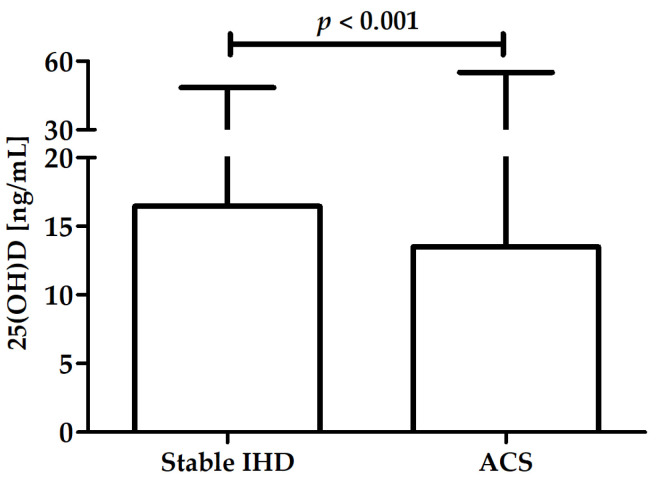
Difference in 25(OH)D between patients with stable IHD and ACS.

A significant difference in 25(OH)D was observed between patients with stable IHD (median: 16.5 ng/mL, range: 4.1–48.4) and ACS (median: 13.5 ng/mL, range: 4.0–55.0) (Figure 2). 

There were significant differences in both SII and SIRI between patients with stable IHD and ACS: patients with ACS presented significantly higher values of both biomarkers (see our previous study [21]).

### 3.5. Factors Associated with ACS Diagnosis

The results of the multivariable logistic regression analysis of factors associated with ACS diagnosis are presented in Table 3. The following factors were associated with ACS diagnosis hypertension and smoking. Patients with hypertension and who smoked actively had higher odds of ACS diagnosis. 

The Hosmer–Lemeshow goodness-of-fit test produced a test statistic of 9.146 (with a *p*-value of 0.330). The AUC of the regression model was 0.632.

## 4. Discussion

This research assessed the correlation between serum vitamin D concentrations with new inflammatory markers—SIRI and SII—in a group of nearly 700 patients undergoing coronary angiography due to suspicion of ACS. This article is a continuation of a project analyzing blood cell counts as biomarkers of subclinical inflammation in IHD [73,74]. The previously presented data revealed that SII and SIRI were significantly higher in patients with diagnosed ACS compared with those in patients with stable IHD [21]. In the present study, we showed that patients diagnosed with ACS had lower serum vitamin D concentrations. In addition, SIRI (but not SII) was significantly correlated with the serum vitamin D concentration in the entire analyzed group. SIRI and SII were both negatively associated with vitamin D levels in patients with ACS but not in the stable IHD group. 

Chronic subclinical inflammation has a major influence on CVD development [75], causing myocardial ischemia–reperfusion injury in ACS [76,77]. The correlation between classic inflammatory markers and the extent of IHD and its complications has previously been shown [10,12,13,78,79]. Therefore, the residual inflammation risk decrease measured with C-reactive protein concentration has been reported to reduce the occurrence of MACE in patients with IHD [80,81,82,83]. 

The innate and adaptive immune response plays a key role in the chronic inflammation of the vessel walls [6]. The mechanisms connecting inflammatory processes with ACS are not fully described; however, recent research indicated the involvement of various immune cells and pro-inflammatory cytokines in plaque destabilization [76]. Neutrophils are involved in atherogenesis and the occurrence of ACS [84], as their count is positively correlated with plaque erosion risk [85], microcirculation vessel injury, and thrombosis risk [86]. Monocytes are an independent predictor of CVD mortality [87], and together with cytokines, proteolytic enzymes, and RO are involved in the development and progression of atherosclerosis [88]. Only lymphocytes have anti-atherosclerotic function [89], as their low number was associated with the progression of atherosclerosis and an increased risk of MACE in patients with ACS [90]. Moreover, activated platelets also contribute to the pathogenesis of ACS, as they have pro-inflammatory and prothrombotic properties [91,92]. 

Among immune cells, monocytes are the most influenced by vitamin D, as vitamin D blocks them from morphing into dendritic cells [93]. Decreased levels of calcitriol are responsible for the cytolytic and pro-inflammatory properties of monocytes [94]. In neutrophils, vitamin D decreases adhesion and aggregation [95]; therefore, its deficiency leads to impaired migration, a decrease in leukotriene B4 synthesis, increased ROS, and pro-inflammatory cytokine production [96]. It also modulates the adaptive response of Treg and Th2 lymphocytes through VDR activation and leads to their activation and increased production of anti-inflammatory cytokines [97,98]. Calcitriol decreases the production of pro-inflammatory cytokines by T1 lymphocytes [99,100], blocks the maturation of B lymphocytes and their transformation into effector B cells, and decreases their expression of MHC-II [101,102]. Furthermore, it has an immunosuppressive effect via the NFκB transcription factor [103]. In vivo research showed that physiological doses of vitamin D inhibited IL-17 production, which is involved in plaque destabilization [104]. Calcitriol has indirect antithrombotic properties—it decreases the expression of the adherence receptor CD62P [105,106] and the concentration of tissue factor in platelets, and it increases thrombomodulin levels [107].

New inflammatory biomarkers linking three types of immune cells—SII and SIRI—attempt to describe the pro- and anti-inflammatory balance and quantify the complex impact of the immune system on atherosclerosis. The results of previous research suggest a correlation between higher values of these markers and the occurrence of ACS [21,23,29]. It is worth noting that significantly lower serum vitamin D concentrations [60] as well as higher levels of both biomarkers [21] were previously found in patients with ACS, which corroborates the data presented in this article. Considering the influence of vitamin D on all cells included in those markers, our data suggest that there is a correlation between vitamin D concentration and SII and SIRI as markers of subclinical inflammation involved in atherogenesis. Recent data describing calcifediol levels and chronic kidney disease as independent factors of calcitriol insufficiency also showed a correlation between decreased vitamin D and other inflammatory markers (C-reactive protein, uric acid, homocysteine, and fibrinogen) in patients with chronic kidney disease [108]. The correlation of SIRI but not SI, in the entire group of IHD patients may be due to SIRI utilizing the number of monocytes, which are more susceptible to vitamin D than the platelets used in calculating SII [93,109]. To our knowledge, this is the first research on the association between vitamin D and SIRI or SII. 

The main limitation of this research is its cross-sectional and observational design, which disables the possibility of causational analysis. This study included a limited number of patients who lived in central Poland. Patients with significantly increased CRP and white blood cell count were excluded from this study; other inflammatory markers (TNF-alpha, IL-6, ferritin) were not measured, however. The influence of comorbidities, smoking status, and prescribed treatment (including statins) was not taken into account. Due to the short half-life of calcitriol, only cholecalciferol was measured. 

In addition to the well-established position of vitamin D in the skeletal system, its role in the pathogenesis of CVD, as well as the correlation between low levels of this hormone and increased cardiovascular risk, has been emphasized [110,111,112]. Recent randomized studies have not provided evidence that vitamin D supplementation is beneficial in reducing CVD mortality [113], as most of the studies have been carried out in patients without the symptoms of vitamin D deficiency [114,115]. However, a recent meta-analysis of 19 observational and 3 randomized studies revealed a negative correlation of serum vitamin D with carotid intima-media thickness (CIMT) and a positive influence of vitamin D supplementation on the decrease in CIMT [116]. Moreover, the non-linear analysis of magnetic resonance imaging performed in UK Biobank showed an L-shaped correlation between genetically conditioned vitamin D concentration and CVD risk [117]. Today, the mechanisms that link deficiency with acute complications of atherosclerosis are still under intensive investigation.

Inflammatory biomarkers that include many mutually interacting factors could provide additional data on the subclinical inflammation that influences atherosclerosis and its complications. The role of vitamin D in the pathogenesis of ACS, its impact on the immune system, and inflammatory markers should be the subject of further well-designed research. 

## 5. Conclusions

In patients undergoing coronary angiography due to suspected ACS, serum vitamin D concentration was correlated with SIRI (but not SII). Vitamin D levels were significantly lower in patients with ACS diagnosed compared with those in patients with stable IHD. Both SII and SIRI were negatively correlated with vitamin D concentrations in patients diagnosed with ACS. The role of vitamin D in complications of IHD and its association with new inflammatory biomarkers requires further large-scale, well-designed research.

## Figures and Tables

**Table 1:** Characteristics of participants.

Variable	Values
N of participants (♂/♀)	443 (63%)/256 (37%)
Age (years)	66.3 (59.4–75.0)
BMI (kg/m^2^)	27.8 (24.9–31.1)
Cause of hospitalization (stable IHD/ACS)	366 (52%)/333 (48%)
Previous MI (yes/no)	269 (38%)/430 (62%)
Total cholesterol (mg/dL)	172.0 (143.5–203.8)
High-density lipoprotein (mg/dL)	47.1 (39.2–57.7)
Low-density lipoprotein (mg/dL)	95.6 (72.2–124.7)
Triglycerides (mg/dL)	113.9 (86.0–158.6)
Hyperlipidemia (yes/no) (*n* = 644)	377 (54%)/267 (38%)
Hypertension (yes/no)	577 (83%)/122 (17%)
Smoking (active/former smoker/no)	195 (28%)/75 (11%)/429 (61%)
Type 2 diabetes mellitus (yes/pre-diabetes/no)	236 (34%)/30 (4%)/433 (62%)
Leukocytes (thousand cells/µL) (*n* = 694)	8.0 (6.5–9.6)
Platelet (mcL)	220 (184–259)
Neutrophils (thousand cells/µL)	4.8 (3.7–6.2)
Monocytes (thousand cells/µL)	0.7 (0.6–0.9)
Lymphocytes (thousand cells/µL)	1.9 (1.6–2.5)
SII	519 (373–774)
SIRI	1.7 (1.1–2.7)
Serum 25(OH)D (ng/mL)	15.1 (10.2–21.3)

Data presented as numbers (%) or medians (interquartile range: 25th–75th percentiles). BMI—body mass index; IHD–ischemic heart disease; ACS—acute coronary syndrome; MI—myocardial infarction.

**Table 2:** Determinants of ln25(OH)D concentration.

Determinants	β (SE)	*p*-Value
Age	−0.05 (0.05)	0.306
Sex (♀/♂)	0.04 (0.05)	0.424
BMI	0.05 (0.05)	0.325
Examination date (May–October/November–April)	−0.14 (0.04)	<0.000
Smoking (no/yes)	−0.09 (0.05)	0.039
Hyperlipidemia (no/yes)	−0.20 (0.04)	<0.000

SE—standard error; BMI—body mass index.

**Table 3:** Multivariable logistic regression analysis of factors associated with ACS diagnosis.

Variables	Category	β	Wald Stat. 95% CI	Odds Ratio (95% CI)	*p*-Value
Age	-	−0.01	2.60 (−0.03–0.00)	0.99 (0.97–1.00)	0.107
BMI	-	−0.02	1.50 (−0.06–0.01)	0.98 (0.94–1.01)	0.219
25(OH)D	-	−0.02	4.62 (−0.05–0.00)	0.98 (0.95–1.00)	0.032
Sex	Men	0.01	0.00 (−0.37–0.40)	1.01 (0.69–1.49)	0.949
Hypertension	Yes	0.62	6.09 (0.13–1.11)	1.85 (1.14–3.02)	0.014
Diabetes	Yes	−0.22	1.23 (−0.62–0.17)	0.80 (0.54–1.19)	0.267
Hyperlipidemia	Yes	0.06	0.10 (−0.32–0.44)	1.06 (0.73–1.56)	0.750
Smoking	Yes	0.50	5.37 (0.08–0.91)	1.64 (1.08–2.49)	0.021
Examination date	May–October	−0.10	0.20 (−0.54–0.34)	0.90 (0.58–1.41)	0.657

## Data Availability

Data can be provided by the corresponding author upon reasonable request.
